# Functional Electrical Stimulation Therapy for Retraining Reaching and Grasping After Spinal Cord Injury and Stroke

**DOI:** 10.3389/fnins.2020.00718

**Published:** 2020-07-09

**Authors:** Naaz Kapadia, Bastien Moineau, Milos R. Popovic

**Affiliations:** ^1^Rehabilitation Engineering Laboratory, The KITE Research Institute, Toronto Rehabilitation Institute-University Health Network, Toronto, ON, Canada; ^2^Rehabilitation Sciences Institute, University of Toronto, Toronto, ON, Canada; ^3^CRANIA, University Health Network and University of Toronto, Toronto, ON, Canada; ^4^The KITE Research Institute, Toronto Rehabilitation Institute-University Health Network, Toronto, ON, Canada; ^5^Institute of Biomaterials and Biomedical Engineering, University of Toronto, Toronto, ON, Canada; ^6^Myant Inc., Toronto, ON, Canada

**Keywords:** functional electrical stimulation, spinal cord injury, stroke, rehabilitation, electrical stimulation, grasping, reaching, arm function

## Abstract

Neurological conditions like hemiplegia following stroke or tetraplegia following spinal cord injury, result in a massive compromise in motor function. Each of the two conditions can leave individuals dependent on caregivers for the rest of their lives. Once medically stable, rehabilitation is the main stay of treatment. This article will address rehabilitation of upper extremity function. It is long known that moving the affected limb is crucial to recovery following any kind of injury. Overtime, it has also been established that just moving the affected extremities does not suffice, and that the movements have to involve patient’s participation, be as close to physiologic movements as possible, and should ideally stimulate the entire neuromuscular circuitry involved in producing the desired movement. For over four decades now, functional electrical stimulation (FES) is being used to either replace or retrain function. The FES therapy discussed in this article has been used to retrain upper extremity function for over 15 years. Published data of pilot studies and randomized control trials show that FES therapy produces significant changes in arm and hand function. There are specific principles of the FES therapy as applied in our studies: (i) stimulation is applied using surface stimulation electrodes, (ii) there is minimum to virtually no pain during application, (iii) each session lasts no more than 45–60 min, (iv) the technology is quite robust and can make up for specificity to a certain extent, and (v) fine motor function like two finger precision grip can be trained (i.e., thumb and index finger tip to tip pinch). The FES therapy protocols can be successfully applied to individuals with paralysis resulting from stroke or spinal cord injury.

## Introduction

Application of functional electrical stimulation (FES) for therapeutic purposes in rehabilitation settings dates back to the 1960’s when Liberson et al. (1961) used an FES system to stimulate the peroneal nerve to correct foot drop by triggering a foot switch, a single-channel electrical stimulation device stimulated the common peroneal nerve via a surface electrode, producing ankle dorsiflexion during the swing phase of gait (Liberson et al., 1961). This led to the first commercially available FES system with surface stimulation electrodes. Since then FES technology has been researched extensively to evaluate its benefits in diverse neurological conditions, and using an array of application techniques (Baldi et al., 1998; Field-Fote, 2001; Popovic et al., 2005, 2011, 2012, 2016; Yan et al., 2005; Frotzler et al., 2008; Griffin et al., 2009; Daly et al., 2011; Kapadia et al., 2011, 2013, 2014a; Giangregorio et al., 2012; Malešević et al., 2012; Martin et al., 2012; Kawashima et al., 2013; Lee et al., 2013; Sadowsky et al., 2013; Ho et al., 2014; Kapadia N. et al., 2014; Popović, 2014; Sharif et al., 2014; Bauer et al., 2015; Howlett et al., 2015; Vafadar et al., 2015; Buick et al., 2016; Cuesta-Gómez et al., 2017; Fu et al., 2019; Straudi et al., 2020). The two common uses of FES are to replace function (i.e., as an orthotic device) and to retrain function (i.e., as a therapeutic device). In this article we will limit ourselves to the therapeutic application of FES.

In the therapeutic application (FES therapy), FES is used as a short-term treatment modality. The expectation is that, after training with the FES system, the patients will be able to voluntarily perform the trained activities without FES (i.e., patients are expected to regain voluntary function). To date, a few high-quality randomized controlled trials have been performed, proving the efficacy of FES therapy over other rehabilitation techniques (Sharififar et al., 2018; Yen et al., 2019). This paucity in multicenter randomized controlled trials and the limited access to systems that can properly deliver FES therapy might have affected its uptake in clinical settings (Ho et al., 2014; Auchstaetter et al., 2016). Fortunately, both these issues are being addressed as new FES systems that are specifically developed for FES therapy are being introduced, as well as large scale multicenter randomized controlled trials are being planned to further confirm the efficacy of this rehabilitation modality. This article will provide readers with the details on how transcutaneous multichannel FES therapy for the upper extremity can be applied in clinical trials and as such the same methodology can be used in clinical practice by physiotherapists and occupational therapists.

The FES methodology discussed here has been developed with the intent to be user friendly, robust and to be able to produce better functional gains than the presently available best-practice rehabilitation techniques. The FES system used in our laboratory is a surface stimulation system with up to 4 stimulation channels that can produce gross motor function as well as precision grips such as two finger pinch grip. However, the methodology of FES application discussed here is pertinent to any multichannel transcutaneous FES device. We have used transcutaneous FES to retrain reaching and grasping in individuals with both spinal cord injury and stroke (Thrasher et al., 2008; Kapadia and Popovic, 2011; Kapadia et al., 2011, 2013; Popovic et al., 2012; Hebert et al., 2017). The results obtained in both patient populations indicate functional improvements after 8–14 weeks of therapy (20–48 h of stimulation). Patients showed reduced dependency on caregivers, and some even became independent in their activities of daily living.

This article will extensively detail how FES was applied in these previously successful clinical trials to retrain reaching and grasping functions in individuals who sustained a spinal cord injury or a stroke.

## Materials and Equipment

The FES system we used was a four channel surface stimulation device consisting of a software, a portable stimulator with a programmed chip card, self-adhesive stimulation electrodes, and various man-machine interfaces, such as push buttons, sliding potentiometers (Mangold et al., 2005), accelerometers (Widjaja et al., 2011), EMG/biofeedback sensor, joysticks (Sayenko et al., 2013), foot switches (Popovic et al., 2001b), gait phase detection system (Pappas et al., 2004) and brain–machine interface (Márquez-Chin et al., 2009). This FES system has been extensively used in clinical trials by researchers both in North America and in Europe. Its unique capability is the ability to program stimulation protocols customized to a patient’s needs in less than 10–15 min.

### Software

The software of our FES system allows one to specify/alter all stimulation parameters: frequency, minimum and maximum intensity, pulse duration, ramp time, synchronization and order of stimulations, type of user interactions and number of repetitions. The sensory, motor, functional and maximum thresholds are set using the continuous stimulation mode where the stimulation frequency and pulse duration are pre-set to values of 40 Hz and 200 μs, respectively.

## Methods

### Clinical Applications

To date, approximately 150 spinal cord injury and 50 stroke patients have been treated using transcutaneous FES in our facilities, ranging from pilot clinical trials to randomized controlled trials. The FES system has been primarily used as a therapeutic device for retraining reaching and grasping. More recently FES was successfully applied to an individual with cervical spondylotic myelopathy to retrain upper extremity function with very promising results (Popovic et al., 2016).

#### Neuroprosthesis for Grasping in Spinal Cord Injury Patients (University Health Network REB # 02-032, REB # 09-007, REB # 09-008)

In case of patients with spinal cord injury the upper extremity retraining program is designed based on the level and extent of injury. For example, in C1–C5 cervical incomplete injuries initially FES might be used to retrain proximal function and then once the patient is able to position their arm in the working space then distal function can be trained. The FES protocols for retraining proximal function in SCI remain the same as stroke (please refer to the next section on stroke for details). In patients with low cervical injury (C6 and below), proximal upper extremity function might be preserved, and FES might then be used to retrain distal function right from the beginning. Also, it is important to note that again based on level of injury patient with SCI may have difficulty with both hand closing and opening and will typically need to be trained for both.

Over the years, various grasping protocols have been identified and designed allowing for a wide variety of grasping patterns to be trained with a great deal of fidelity. Currently, the grasping patterns that can be successfully retrained using a transcutaneous multi -channel FES system are:

(1) Palmar Grasp (holding a ball)
(2) Lateral Grasp (holding a tray)
(3) Tripod grip (thumb, index, and middle finger: holding a pen)
(4) Two finger opposition (thumb and index finger: holding a peg)
(5) Lateral Pinch (thumb and index finger: holding a credit card)
(6) Two finger lateral pinch (index and middle finger: smoker’s grip)
(7) Lumbrical grip (all four fingers with the thumb: holding a closed book).

It is important to mention that FES therapy has the capability to help stroke and spinal cord injury patients relearn how to voluntarily perform all of the above grasps bilaterally and simultaneously, using surface FES system.

We have conducted a number of clinical studies using this FES technology the most recent one being a randomized controlled trial in incomplete cervical SCI patients (Popovic et al., 2011). Individuals allocated to the intervention group in this trial received FES stimulation protocols specifically designed for their upper extremity functional deficits. Individualized stimulation sequences were developed for each patient. The commonly trained grasping patterns were power and precision grasp where power grasp was used mainly to grasp larger everyday objects and the precision grip was used mainly to manipulate smaller objects. Power grasp was generated by partly flexing the fingers and the thumb in flexion and slight opposition. Lateral pinch was generated by fully flexing the fingers followed by the thumb flexion. Muscles that were stimulated during therapy were the following:

• Wrist flexors: flexor carpi radialis and flexor carpi ulnaris;
• Wrist extensors: extensor carpi radialis (longus and brevis) and ulnaris;
• Finger flexors: flexor digitorum superficialis and flexor digitorum profundus;
• Finger extensors: extensor digitorum;
• Thumb abductors: median nerve, or abductor pollicis brevis and longus;
• Thumb flexors: flexor pollicis brevis and flexor pollicis longus;
• Thumb oppositors: opponens pollicis;
• Metacarpophalangeal flexors and interphalangeal joint extensors: lumbricals.

The FES protocol allowed for individuals with little to no voluntary movement at the wrist and fingers to be able to perform simple tasks while being stimulated with the FES. This is what differentiates FES from other therapies. In the early stages of FES therapy, all the movements were performed with the help of FES. The treatment plan and instruction to participants were as follows:

(1) “Imagine hand opening” (or any movement that the therapist would like to train).
(2) “Try to perform the movement using your own muscle strength.”
(3) After trying for about 10 s: “Now, try to perform the movement with the help of FES.”

Hence, emphasis was put on participants voluntarily attempting the movement while being stimulated with the FES. During therapy when the participants started showing an ability to voluntarily contract certain muscle groups FES for those muscle groups was reduced to a minimum and gradually withdrawn completely. The available channel was then used on other muscle groups that were still weak and needed to be trained. The order in which muscle groups were sequentially “reactivated” was subject-dependent. FES was always delivered while the participants were performing functional tasks, such as grasping a mug, pouring water, holding a pen, etc.

The distinctiveness of this intervention is that FES is not primarily intended for muscle strengthening. Instead, it is used to retrain the neuromuscular system to execute tasks that it is unable to carry out voluntarily. Movements were performed against gravity and sometimes against light manual resistance. The number of repetitions was determined based on individual participant’s strength and endurance. In general, all participants spent 30–45 min out of 1-h session performing activities of daily living with FES. The stimulation parameters used were the following: (a) balanced, biphasic, current regulated electrical pulses; (b) pulse amplitude from 8 to 50 mA (typical values 15–30 mA); (c) pulse width 250 μs; and (d) pulse frequency 40 Hz (Popovic et al., 2011). During the intervention, the therapist, at their discretion, adjusted the placement of electrodes and guided the hand movements. The therapist ensured that the movements were functional. Occasionally FES would be combined with conventional rehabilitation strategies including strengthening exercises, stretching exercises, etc.

#### Neuroprosthesis for Grasping in Stroke Patients (University Health Network REB # 02-032).

The most important difference between FES training in spinal cord injury and stroke patients; is that stroke patients have difficulty opening their hand as they often exhibit flexor synergy and high levels of tone in the finger flexors. In stroke patients therefore, the focus of the therapy is on hand opening and relaxing the fingers. In spinal cord injury patient’s the focus of the FES therapy is on finger flexion and grasping tasks as weakness of the finger flexors is a bigger problem. Below are the methods of FES application in clinical trials conducted in individuals who suffered a stroke (Popovic et al., 2005; Thrasher et al., 2008; Kapadia et al., 2013).

For individuals allocated to the FES therapy group, treatment began by proximal shoulder muscle training. The muscles that were stimulated were deltoid, biceps, and triceps. Typically, participants would recover proximal function first. As soon as they gained functional strength in the proximal muscles, FES for those muscles would be discontinued and applied to distal muscles of the forearm and hand. The most difficult and time-consuming task was to train voluntary extension of the fingers. This is crucial to be able to get one’s hand around the objects that need to be manipulated. Once the participants were able to successfully open their hand with FES assistance, low amplitude stimulation of the finger flexors was used to signal hand closing. Stimulation parameters used to stimulate the muscles and nerves were the same as used for individuals with spinal cord injury (See section on “Neuroprosthesis for Grasping in Spinal Cord Injury Patients).

In the early stages of the treatment, the arm/hand tasks were performed predominantly with the help of FES. As participants showed improvement stimulation was gradually reduced to a minimum and eventually phased out. Typical treatment session lasted for about 45 min, including the donning and doffing of electrodes. During all FES sessions the physiotherapist guided the movements and provided assistance as appropriate to carry out the intended movement in as close to physiological manner as possible.

Over the years the FES-reaching protocols have expanded to cover various functional reach patterns:

(1) Sideways reaching
(2) Sideways reaching with hand opening
(3) Forward reaching and retrieving
(4) Forward reaching and retrieving with hand opening
(5) Reaching over opposite shoulder
(6) Reaching over opposite shoulder to forward reaching to sideways reaching
(7) Reaching over opposite knee
(8) Hand to mouth

All of these protocols can easily be paired with the FES-grasping protocols for the spinal cord injury population to train reaching and grasping together.

### Practical Considerations for Therapist

In most of the clinical trials, FES sessions of 45–60 min were delivered 3–5 days a week, for 8–16 weeks, for a total of about 40 sessions. In our clinical experience, we found that patients are able to tolerate a maximum of one 60 min session per day and within the session typically we are able to stimulate one movement pattern for approximately 10–15 repetitions before fatigue sets in, however, it is important to note that this frequency is individual based and may vary based on extent of injury, chronicity and status of neuro-muscular system. Self-adhesive surface stimulation electrodes were used during therapy. All the patients were treated by registered physiotherapists or occupational therapists. In all instances, each phase of the FES was triggered by the treating Physiotherapist or Occupational therapist using a push button. All FES sessions incorporated functional tasks during FES sessions. All FES training was in combination with conventional physiotherapy or occupational therapy techniques selected based on individual patient needs. Also, irrespective of the population, patients were required to concentrate and actively make an attempt to carry out the movement while being assisted by FES, as described above.

The stepwise directions to conduct an upper extremity FES training session with a transcutaneous multi-channel FES device are as follows:

(1) *Identify the functions to be trained* (reaching and/or grasping).
(2) *Select the order of the tasks to be re-trained:* typically, start with gross motor tasks (proximal muscles) in early stages of therapy followed by fine movement control (distal muscles).
(3) *For each task identify the muscles to be stimulated: at any given time either only simple* reaching or grasping tasks such as touching mouth or palmar grasp can be trained or more complex tasks such as reaching + grasping can be trained based on number of channels available for stimulation.
(4) *First identify the optimal electrodes positioning*: For a given function, find the motor point; the electrode position where a maximal contraction is obtained with minimum stimulation current delivered. We recommend finding the motor point using a smaller electrode, by trying several positions on the bulk of the muscles to be stimulated. This allows for finding an electrode position with minimal secondary and unintentional stimulation of other muscles and/or nerves. Once you find the optimal electrode position(s) for a muscle, mark it with a pen/marker, and identify position(s) for other muscles.
(5) Apply self-adhesive electrodes over the motor points of the muscles identified.
  *Note:* In case one has a stimulator that has galvanically isolated stimulation channels, one can apply the following: all electrodes on one aspect of the forearm can be “grounded” using a single return/anode electrode, i.e., all muscles on the palmar aspect of the forearm can be grounded using one electrode just proximal to the ventral aspect of the wrist joint and similarly all electrodes on the dorsal aspect of the forearm can be grounded using one electrode over the dorsal aspect of the wrist. If the stimulator does not have galvanically isolated stimulation channels one should not use this “common ground” strategy.
  If you use non-alternate and asymmetrical pulses waveform (with the negative depolarizing pulse always on the same electrode, and the positive balancing pulse at a lower amplitude), then you will have an “active” electrode to be positioned on the motor point, and a “passive” or return/anode electrode under which there is no effective stimulation (setting typically used for smaller muscles). If you use alternate and/or symmetrical pulse waveform, then both electrodes are “active” and will trigger contractions similarly (setting typically used for larger muscles). The choice between one or two active electrode(s) is based on the muscle size (one active electrode is preferable where there is no space on the bulk of the muscle to position two electrodes). Also, having a single “active” electrode ensures greater specificity of the muscle and muscle volume that is stimulated.
(6) *Identify and record the different stimulation thresholds:* Identify sensory threshold (when the patient feels the current for the first time), motor threshold (when a palpable or a visible contraction is produced), functional threshold (when the desired functional movement is produced) and maximum threshold (beyond which the patient does not tolerate an increase in current amplitude).
  *Note:* It is important to define the thresholds with the same current characteristics (pulse width and frequency) as the one used during FES therapy, because it has an impact on the comfort and efficiency of the stimulation.
(7) Explain to the patient what to expect when the FES in turned on
  Example: “First your hand will close and then it will open.”
(8) *Turn on the stimulator and adjust the current intensities for all muscles to the levels determined previously* (intensity should not exceed the determined maximum threshold). Trigger the FES protocol a few times so the patient has a clear understanding of what to expect with each phase of FES. Once the patient has a clear understanding of the protocol, select the functional object to be used during training. If needed, assist the patient to bring their hand close to/around the object to be manipulated.
(9) Instruct the patient that she/he has to make an active attempt to perform the intended movement.
  *Example:* For a grasp/release task, ask the patient to close the hand to grasp the object and, after the patient has attempted for about 5–10 s, assist with FES. Once the patient is able to grasp the object with assistance from FES, complete the functional task, for example transfer object from point A to point B. Following successful object transfer, instruct the patient to release the object and after about 5–10 s of the patient unsuccessfully attempting to release the object trigger the FES sequence for hand opening.
(10) *Repeat this protocol 10–15 times.* Then, select another protocol and perform the next task for 5–7 min or as appropriate for that task. Execute 3–6 different protocols in a 1-h session, with active stimulation for 30–40 min (depending on patient’s fatigue and therapist’s expertise with the system). The 1-h therapy duration includes positioning of the electrodes and all relevant preparations for therapy initiation and therapy completion.
(11) Rest time should be given when the patient asks for it and/or when muscle fatigue sets in.
(12) When the therapy is completed, turn off the stimulator, remove the electrodes and inspect the skin underneath for any redness.
  *Note:* Occasionally redness may be present from the electrode sticking on the skin, but it should dissipate in less than 24 h. Ask patient to monitor area and re-inspect at the next session.

The selection of stimulation sequences is done based on clinical assessments which typically include use of standardized assessment tools like Graded Redefined Assessment of Strength, Sensibility and Prehension, Toronto Rehabilitation Institute- Hand Function Test and Spinal Cord Independence Measure Self-care Sub-scores in spinal cord injury (Popovic et al., 2011) and Action Research Arm Test and Fugl Meyer assessment – upper extremity scores in stroke (Hebert et al., 2017).

### Limitations

There are certain limitations to this technology. The limb muscles that are intended for FES treatment have to be accessible for placement of the stimulation electrodes (Popovic et al., 2001a). There should not be a major degree of lower motor neuron injury or nerve-root damage of the stimulated muscle. In a number of patients with spinal cord injury, there may be a variable amount of peripheral nerve damage (Doherty et al., 2002) (motoneurons and nerve-roots) that restricts the application of FES. Also, the patient has to be cognitively able to follow the instructions and actively participate in the therapy process. The patient should not have any contraindications for FES application like metal implants at the site of stimulation, pace-maker, open wound or rash at the site of electrode placement, uncontrolled autonomic dysreflexia, etc.

Besides, with programmable surface stimulation devices, one would need an inter-professional team including bio medical engineers who are proficient in programming the stimulation protocols. This programming limitation may not apply to the more sophisticated newer FES systems. Presently there are commercially available FES systems that can deliver FES therapies discussed in this article. The reader is encouraged to find a device that delivers FES therapies and is approved by the local regulatory body. Systems that do not have neuroplasticity and neuromodulation in their indication for use defined by the regulatory body should be avoided, as these stimulators are for muscle strengthening and improving range of motion, and not for FES therapy discussed in this article.

## Results

To date, in our laboratory transcutaneous FES therapy has been successfully applied to over 200 patients with either stroke or spinal cord injury. Based on the outcomes of these studies, it can be said that short duration FES therapy combined with conventional occupational therapy and physiotherapy has the ability to produce positive changes in these patients (Popovic et al., 2005, 2011, 2016; Thrasher et al., 2008; Kapadia and Popovic, 2011; Kapadia et al., 2013). The underlying mechanism responsible for these changes include central modulation effects. Stimulation induces cortical plasticity by modulating the ascending pathways through the Ia muscle fiber afferents (Chipchase et al., 2011). Additionally, somatosensory inputs to the motor cortex are essential for motor learning and control, and play critical roles in the motor recovery process (Vidoni et al., 2010; Pan et al., 2018). Stimulation above the motor threshold increases excitability of corticomotor pathway by activating sensory axons and recruiting synaptic motoneurons and motor reflex (Chipchase et al., 2011). FES therapy in combination with conventional PT and OT techniques harnesses the benefits of neuroplasticity thereby improving function and enhancing participant independence with activities of daily living.

**FIGURE 1:**
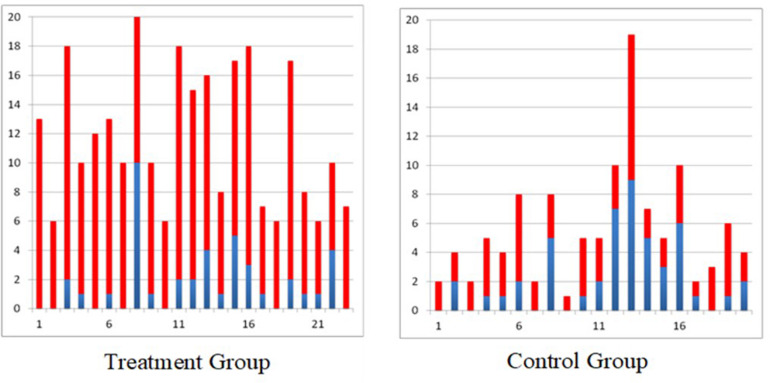
Self-care Spinal Cord Independence Measure scores for all individuals with incomplete sub-acute spinal cord injury (blue bar indicates score at baseline and red bar indicates gain after 40 × 1 h therapy, treatment group received functional electrical stimulation and control group received conventional PT/OT).

In the randomized controlled trial carried out in individuals with subacute (<6 months post injury) incomplete traumatic C3–C7 spinal cord injury, it was found that the individuals who received 40 h of FES therapy had far greater improvements on the Self Care Sub-scores of the Functional Independence Measure and Spinal Cord Independence Measure as compared to individuals who received 40 h of conventional occupational therapy (Popovic et al., 2011). These gains were retained, or further improvement was observed, in the FES therapy group at the time of 6 months follow up assessment (Popovic et al., 2011). To date we have obtained similar results in all individuals with sub-acute incomplete spinal cord injury who received 40 h of FES therapy (Figure 1).

**FIGURE 2:**
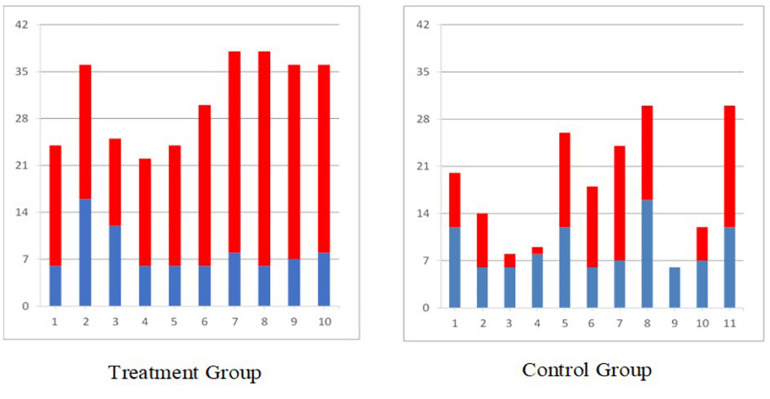
Self-care Functional Independence Measure scores for all individuals with sub-acute stroke (blue bar indicates score at baseline and red bar indicates gain after 40 × 1 h therapy, treatment group received functional electrical stimulation and control group received conventional PT/OT).

Similar results were obtained in the randomized controlled trial carried out in individuals with acute (2–7 weeks post) severe stroke with a total arm and hand score no more than 2 on the Chedoke McMaster Stages of Motor Recovery (less than 15 points on Fugl Meyer Assessment Upper Limb Sub-score) (Thrasher et al., 2008; Hebert et al., 2017; Marquez-Chin et al., 2017). The individuals who received 12–16 weeks of FES therapy for the arm and hand had statistically better improvement on the Self-care sub-score of the Functional Independence Measure (Figure 2), Fugl Meyer Assessment, Barthel Index, and Chedoke McMaster Stages of Motor Recovery as compared to individuals who received conventional occupational therapy and physiotherapy for the same duration. Detailed results of this study are published elsewhere.

In another clinical trial in chronic severe pediatric stroke population (Kapadia N. et al., 2014), where all four participants received a total of 48 h of FES therapy, statistically significant improvements were observed on the Quality of Upper Extremity Skills Test as well as on various sub components of the Rehabilitation Engineering Laboratory Hand Function Test (this is the Toronto Rehabilitation Institute- Hand Function Test with a scoring system adapted for stroke).

## Discussion

Short duration multichannel surface FES is a viable and safe treatment modality that can be successfully applied in patients with neurological conditions. It is important to note that we did not formally investigate safety and feasibility in our clinical trials mainly because transcutaneous FES has been applied in clinical trials for over 5 decades now without any reports of major adverse events. However, given that we have applied FES to over 200 patients over the past 15+ years we are able to confidently say that transcutaneous FES is both safe and feasible. Across all of our clinical trials we did not encounter any serious adverse events and we have been able to successfully retain our study participants for the duration of the research therapy. Discussed here is an in-depth application of transcutaneous multi-channel FES therapy of the upper extremity in spinal cord injury and stroke patients. In order to obtain maximum benefits of this therapy there are some general points to keep in mind.

The goal of this manuscript is not to explore the mechanism of improvement in individuals with stroke and spinal cord injury following FES as this is a methods paper and as such these mechanisms have been widely discussed in literature (Quandt and Hummel, 2014; Hara, 2015; Luo et al., 2020; Marquez-Chin and Popovic, 2020). We do, however, recommend some basic principles of FES application on the widely accepted belief that mechanism of improvement with this therapy is based on the principles of neuroplasticity (Nagai et al., 2016). First and foremost it is strongly recommended that therapy should be started as soon as the medical condition of the patient is stabilized, i.e., preferably in the acute or sub-acute phase post-injury. Secondly, active participation of the patient during treatment is critical. Along with the FES, patients have to make an active attempt to execute the target movement. Third, the movements carried out should be functional and should follow a physiological pattern as closely as possible (movements similar to those of able-bodied individuals). Fourth, therapy should be combined with conventional rehabilitation modalities (example: stretching and strengthening) to reap maximum benefits. Lastly, while no specific dosing study has been conducted, our group recommends delivering at least three 1 h sessions per week. However, our group does not recommend more than one session per day, as this often exhausts the patient and prevents them from actively participating in the second session. In total, at least 20 sessions are needed to obtain clinically relevant changes, however, it is often recommended that patients have 40 or more hours of therapy to maximize outcomes and experience greater gains in function.

It should be noted that, in certain very acute or chronic spinal cord injury cases, a strengthening phase is necessary prior to the functional training phase because the muscles are minimally responsive to stimulation at first (Popovic et al., 2002) due to initial spinal shock (Galeiras Vázquez et al., 2017) or due to long-term disuse (Popovic et al., 2002).

It is important to bear in mind that although FES therapy has not been extensively tested in individuals with cervical complete spinal cord injury, those that have been trained with the system have shown remarkable improvements that were much more profound than those produced with conventional therapy (Popovic et al., 2006). This evidence merits conducting more comprehensive clinical trials with FES therapy in cervical complete spinal cord injury patients.

In conclusion, the most attractive feature of multichannel surface stimulators is that they are non-invasive, often programmable and allow for various muscles/muscle groups to be stimulated simultaneously in physiological patterns. They have a high level of fidelity and are able to produce global upper-limb motions as well as fine finger movements like two pinch grip (thumb and index finger) and tripod grip (thumb, index, and middle finger) using surface stimulation electrodes.

The specific surface stimulator used in our clinical studies, is not widely available any longer, however, the methodological considerations discussed above remain the same irrespective of the type of stimulation device. Any stimulator that can produce protocols discussed in this article can be used for FES therapy. Although the new stimulators used for the FES therapy come with guidelines for locating motor points, therapists should be mindful that motor points can anatomically vary between individuals. If required, the first session should be dedicated to finding correct stimulation points, after which these can be marked down for future sessions.

As important as it is to assist weak muscles with FES during execution of functional tasks, it is equally important that once functional voluntary strength is recovered (at least 3/5 on Manual Muscle Testing), stimulation is withdrawn from those muscles and the patient is encouraged to voluntarily control the muscles themselves. The available FES channels can then be applied to other weaker muscle groups that still need retraining. In some cases, with severe spasticity, manual stretching of the tight muscles prior to stimulation may yield better results.

## Data Availability Statement

The datasets generated for this study are available on request to the corresponding author.

## Ethics Statement

The studies involving human participants were reviewed and approved by Research Ethics Board, Toronto Rehabilitation Institute-University Health Network. The patients/participants provided their written informed consent to participate in this study.

## Author Contributions

NK, MP, and BM were responsible for the concept and writing of the manuscript and had read and approved the manuscript. All authors contributed to the article and approved the submitted version.

## Conflict of Interest

MP is a shareholder in company MyndTec Inc. Technology and the results presented in the document, except for study “D. A. Hebert, J. M. Bowen, C. Ho, I. Antunes, D. J. O’Reilly, and M. Bayley,” “Examining a new functional electrical stimulation therapy with people with severe upper extremity hemiparesis and chronic stroke: A feasibility study,” “British Journal of Occupational Therapy, pp:1–10, 2017,” have been produced before the company created its first product. MyndTec did not participate in any aspect of data generation, data acquisition, data processing, data interpretation, manuscript preparation and it did not financially support any aspect of this project or the studies that were discussed in this article. BM is affiliated with Myant Inc., a company that develops electrical stimulation garments. Myant Inc. was not involved in the preparation of this manuscript. The remaining author declares that the research was conducted in the absence of any commercial or financial relationships that could be construed as a potential conflict of interest.
